# Evaluating the 1-Minute Sit-to-Stand Test for Predicting Postoperative Complications after Video-Assisted Thoracic Surgery Lung Lobectomy

**DOI:** 10.5761/atcs.oa.25-00144

**Published:** 2025-09-11

**Authors:** Noriyoshi Sawabata, Masatsugu Hamaji

**Affiliations:** 1 Department of Thoracic Surgery, Kawanishi City Medical Center, Kawanishi, Hyogo, Japan; 2 Department of Thoracic and Cardiovascular Surgery, Nara Medical University, Kashihara, Nara, Japan; 3 Department of Diagnostic Pathology, Nara Medical University, Kashihara, Nara, Japan

**Keywords:** 1-minute sit-to-stand test, video-assisted thoracic surgery, lung resection, preoperative risk assessment, postoperative complications

## Abstract

**Purpose:**

This study aimed to determine whether the 1-minute sit-to-stand test (1-min STST) can be a predictor of postoperative complications following video-assisted thoracic surgery (VATS) lung lobectomy.

**Methods:**

This retrospective cohort study included 152 patients who underwent VATS lobectomy. Preoperative evaluations included pulmonary function tests, the bendopnea test, and the 1-min STST. The predictive value of these assessments for postoperative complications, graded by the Clavien–Dindo (C–D) classification, was analyzed using logistic regression and receiver-operating characteristic curves.

**Results:**

For predicting C–D grade II or III complications, a 1-min STST repetition count of ≤20 had an area under the curve (AUC) of 0.70, with 90% sensitivity and 46% specificity. For predicting C–D grade III complications, a repetition count of ≤15 showed an AUC of 0.72 (95% confidence interval [CI], 0.39–1.00), with 97% sensitivity and 60% specificity. In multivariate analysis for C–D grade III complications, a lower 1-min STST repetition count was a significant predictor (p <0.01).

**Conclusion:**

The 1-min STST shows potential as a simple tool for preoperative risk stratification in patients undergoing VATS lobectomy.

## Introduction

Pulmonary resection carries an inherent risk of postoperative morbidity and mortality, particularly in patients with pre-existing cardiorespiratory impairment.^1,2)^ Recent advancements in minimally invasive surgery have increased eligibility for lung resection among older patients, highlighting the need for precise preoperative risk assessments to optimize patient outcomes.^3)^

Traditionally, exercise tolerance has been evaluated using tests such as the stair-climbing test and the 6-minute walk test (6MWT).^4,5)^ While valuable, these assessments can be time-consuming and require specific resources, making them less feasible for rapid evaluation in a busy outpatient clinic setting. Consequently, there is growing interest in simpler, quicker functional tests.

The 1-minute sit-to-stand test (1-min STST) has emerged as a practical tool that measures physical endurance and strength, providing valuable insight into a patient’s functional reserve.^6)^ A systematic review by Bohannon and Crouch established that the 1-min STST is a practical, reliable, valid, and responsive measure of exercise capacity, particularly where space and time are limited.^6)^ Its validity is supported by strong correlations with 6MWT distance in patients with pulmonary diseases such as pulmonary arterial hypertension.^7)^

Recent studies have specifically investigated the 1-min STST for predicting postoperative outcomes after lung resection. A prospective study by Boujibar et al.^8)^ found that a threshold of <20 repetitions on the 1-min STST predicted complications (Clavien–Dindo [C–D] grade ≥2) with an excellent area under the curve (AUC) of 0.85. Furthermore, a multicenter study by Quadflieg et al.^1)^ proposed a threshold of ≤22 repetitions to identify patients at risk, with an AUC of 0.71.

This study aimed to determine whether the 1-min STST can serve as a predictive factor for postoperative complications in our cohort of patients undergoing video-assisted thoracic surgery (VATS) lung lobectomy, thereby adding to this growing body of evidence.

## Materials and Methods

### Ethical approval

This research was approved by the Medical Ethics Committee of Nara Medical University (Approval No. 3929), where this study was conducted, and was performed in accordance with the ethical standards of the Declaration of Helsinki. As this study utilized only existing information from medical records, an opt-out methodology was employed instead of obtaining individual informed consent. Information about the research was made publicly available, and patients were given the opportunity to decline participation. This procedure was approved by the Medical Ethics Committee of Nara Medical University.

### Study subjects and eligibility criteria

The study subjects included patients who visited the Department of Thoracic Surgery at Nara Medical University Hospital between January 1, 2022 and December 31, 2023, for surgical treatment of lung cancer. Inclusion criteria were: (i) a clinical diagnosis of lung cancer for which lobectomy was the standard treatment, and (ii) completion of a preoperative bendopnea test and 1-min STST during outpatient evaluation. Patients who underwent thoracotomy, a procedure other than lobectomy, or who were otherwise deemed unsuitable by the principal investigator were excluded.

These preoperative functional assessments were not conducted specifically for this research, but are routinely performed as part of the standard preoperative evaluation protocol for all patients undergoing pulmonary resection in our Department of Thoracic Surgery.

**Fig. 1:**
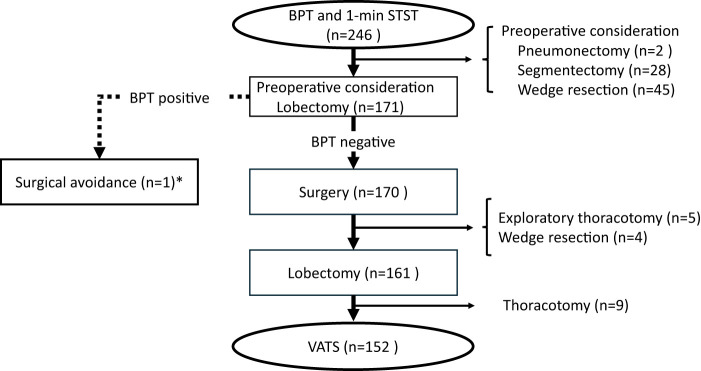
Patient selection flowchart. From the initial 246 patients who underwent preoperative functional testing, 152 patients who underwent VATS lobectomy were included in the final analysis. BPT: bendopnea test; 1-min STST: 1-minute sit-to-stand test; VATS: video-assisted thoracic surgery

While the bendopnea test was used for initial clinical screening as shown in our patient selection flowchart (**Fig. 1**), the primary objective of this study was to specifically evaluate the predictive value of the 1-min STST. Therefore, the results of the bendopnea test were not included as an analytical variable for predicting the primary endpoint.

### Bendopnea test

The bendopnea test was performed as described by Thibodeau et al.^9)^. Patients were asked to bend forward at the waist while seated, and the presence of dyspnea was recorded.

### 1-min STST

The 1-min STST was conducted following a standardized procedure.^6,10)^ Participants were instructed to sit in an armless chair of standard height (45–48 cm) with their feet flat on the floor. They were asked to stand up completely and sit back down as many times as possible within 1 min, without using their hands for support. The number of completed sit-to-stand cycles was recorded, and peripheral capillary oxygen saturation levels (SpO_2_) were monitored to detect exercise-induced desaturation.

### VATS

All surgeons were certified specialists by the Japanese Association for Thoracic Surgery. Surgery was performed in the lateral decubitus position with single-lung ventilation, generally using a 3- or 4-port approach.

### Statistical analysis

Receiver-operating characteristic curves were used to determine optimal thresholds for continuous variables to predict complications, with an AUC of ≥0.60 considered potentially significant. Optimal thresholds were determined using Youden’s index. Univariate and multivariate logistic regression analyses were performed to identify predictors of postoperative complications. Variables with a p-value <0.05 in the univariate analysis were considered for inclusion in the multivariate model. All statistical analyses were performed using EZR (Easy R) software (Saitama Medical Center, Jichi Medical School, Saitama, Japan).^11)^

## Results

### Patient cohort

Of 246 patients who underwent preoperative testing, 152 who had VATS lobectomy were included in the final analysis (**Fig. 1**).

### Patient characteristics

A total of 152 patients underwent VATS lobectomy. Patient characteristics are detailed in **Table 1**. The median age was 73 years, and 67.1% were male. Most patients (96.7%) had a performance status of 0, and 76.3% had comorbidities. The mean percent predicted forced expiratory volume in 1 second (%FEV1) was 90.6%. The results of the 1-min STST are summarized in **Table 2**.

**Table 1:** Characteristics of patients who underwent lobectomy using VATS

	n	%
Total	152	100
Sex		
Male	102	67.1
Female	50	32.9
Age		
Minimum	48	
Maximum	87	
Median	73	
Mean ± S.D.	71.7 ± 8.3	
Height, mean ± S.D.	162.3 ± 8.2	
Weight, mean ± S.D.	61.6 ± 11.0	
BMI, mean ± S.D.	23.3 ± 3.3	
Performance status		
0	147	96.7
1	4	2.6
2	1	0.7
Smoking (BI)		
0	43	28.3
<200	3	2.0
200<, <600	19	12.5
600<	85	55.9
Missing	2	1.3
VC, mean ± S.D.	3093.8 ± 638.9	
%VC, mean ± S.D.	96.0 ± 13.9	
FEV1, mean ± S.D.	2472.6 ± 548.8	
%FEV1, mean ± S.D.	90.6 ± 16.2	
Comorbidity		
Any	116	76.3
Hypertension	69	19.1
Diabetes	18	11.8
Other malignancy	16	10.5
Interstitial pneumonia	15	9.9
Ischemic heart disease	12	7.9
Cerebrovascular accident	8	5.2
%VC <70%	1	0.7
%FEV1 <70%	14	9.2
Clinical stage		
I	127	83.5
II	22	14.5
IIIA	3	2.0
Pathological stage		
I	117	76.9
II	22	14.5
IIIA	12	7.9
IIIB	0	0
IV	1	0.7
Pathological diagnosis		
Adenocarcinoma	106	69.7
Squamous cell carcinoma	33	21.7
Others	13	8.6
Operation		
RUL	60	39.5
RML	10	6.6
RLL	43	28.3
LUL	23	15.1
LLL	16	10.5
OP time (hours), mean ± S.D.	2.9 ± 1.7	
Blood loss (mL), mean ± S.D.	13.8 ± 65.1	

S.D.: standard deviation; BI: Brinkman Index = (number of cigarettes smoked per day) × (number of years of smoking); VC: vital capacity; FEV1: forced expiratory volume in 1 second; RUL: right upper lobe; LML: left middle lobe; RML: right middle lobe; RLL: right lower lobe; LUL: left upper lobe; LLL: left lower lobe; BMI: body mass index; OP: operation

**Table 2:** Results of 1-minute sit-to-stand test

	Repetition	Depression of SpO_2_
Minimum	8	−2
Maximum	41	10
Median	25	1
Mean ± S.D.	26.0 ± 5.8	1.6 ± 1.9

SpO_2_: peripheral capillary oxygen saturation; S.D.: standard deviation

### Postoperative complications

**Fig. 2:**
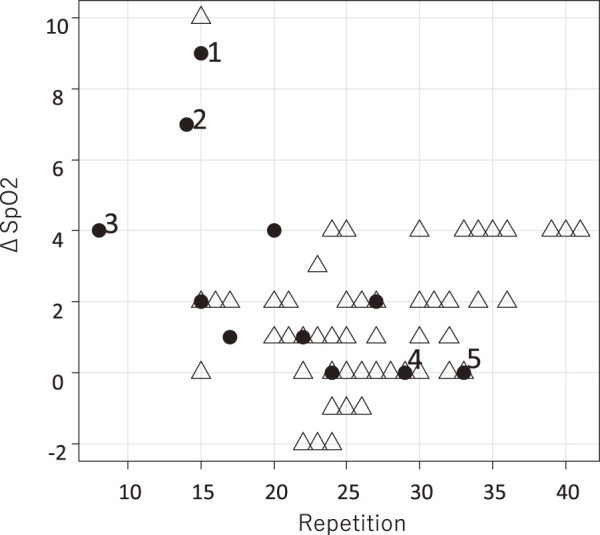
Scatter plot of 1-min STST results and postoperative complications. The white triangle (Δ) indicates cases with no postoperative complications of C–D grade II or III; the black circle (●) indicates a complication of C–D grade II; and the black circles with numbers indicate cases with postoperative complications of C–D grade III. 1, 2: Acute exacerbation of interstitial pneumonia; 3: pneumonia; 4: acute exacerbation of peripheral arterial disease; 5: cerebral infarction. ΔSpO_2_, negative change in peripheral capillary oxygen saturation levels. C-D grade: Clavien–Dindo grade; 1-min STST: 1-minute sit-to-stand test

Postoperative complications occurred in 13 patients (8.6%), as detailed in **Table 3**. Five patients developed C–D grade III complications: acute exacerbation of interstitial pneumonia (n = 2), pneumonia (n = 1), acute exacerbation of peripheral arterial disease (n = 1), and cerebral infarction (n = 1). All patients survived to discharge. The relationship between 1-min STST results and complications is shown in **Fig. 2**.

**Table 3:** Postoperative complications

Calvine–Dindo level	Complication	No.
II	Alveolar-pleural fistula	5
	Atelectasis	2
	Chylothorax	1
III	Acute exacerbation of interstitial pneumonia*	2
	Pneumonia**	1
	Acute exacerbation of peripheral arterial disease***	1
	Cerebral infarction****	1

* Patient’s number 1, 2 in **Fig. 2**. ** Patient’s number 3 in **Fig. 2**. *** Patient”s number 4 in **Fig. 2**. **** Patient’s number 5 in **Fig. 2**.

### Predictors of postoperative complications

**Fig. 3:**
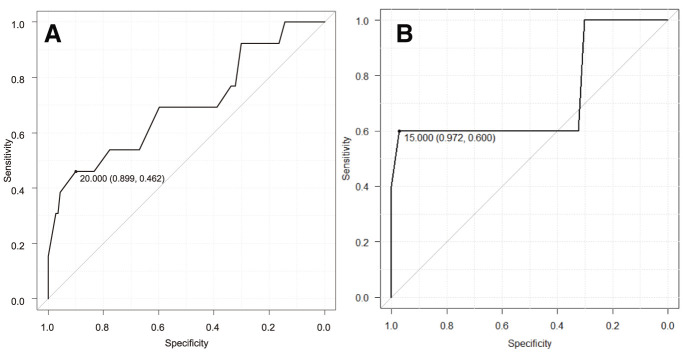
ROC curves for the 1-min STST in predicting postoperative complications. (**A**) ROC curve for predicting C–D grade II or III complications. A 1-min STST repetition count of ≤20 yielded an area under the curve AUC of 0.70. (**B**) ROC curve for predicting C–D grade III complications. A 1-min STST repetition count of ≤15 showed an AUC of 0.72. 1-min STST: 1-min sit-to-stand test; C–D: Clavien–Dindo; ROC: receiver-operating characteristic; AUC: area under the curve

The analysis of postoperative complication prediction is summarized in **Table 4**. For predicting C–D grade II or III complications, a 1-min STST repetition count of ≤20 had an AUC of 0.70, with 90% sensitivity and 46% specificity (**Fig. 3A**). In multivariate analysis, both 1-min STST repetitions (p = 0.02) and %FEV1 (p = 0.03) were significant predictors. For predicting C–D grade III complications, a 1-min STST repetition count of ≤15 had an AUC of 0.72 (95% confidence interval [CI], 0.39–1.00), with 97% sensitivity and 60% specificity (**Fig. 3B**). In multivariate analysis, only the 1-min STST repetition count remained a significant independent predictor (odds ratio per 1-repetition increase, 0.035; 95% CI, 0.00043–0.28; p <0.01).

**Table 4:** Analysis of postoperative complication prediction

	AUC	95% CI	Threshold	Sensitivity	Specificity	n*	Univariate analysis			Multivariate analysis		
							RR	95% CI	p-Value	RR	95% CI	p-Value
C–D II or III												
1-min STST repetition^$^	0.70	0.52–0.87	20	0.90	0.46	120	0.27	0.83e−1– 0.86	0.03	4.43	1.31–15.00	0.02
Depression of SpO_2_	0.53	0.34–0.72										
%VC	0.52	0.32–0.73										
%FEV1	0.69	0.54–0.84	92.43	0.46	0.85	118	0.20	0.43e−1– 0.94	0.04	5.79	1.20–28.00	0.03
Age	0.67	0.55–0.80	74	0.58	0.68	57	2.94	0.91–9.47	0.07			
BMI	0.63	0.46–0.80	23.15	0.53	0.77	78	0.39	0.12–1.33	0.13			
Comorbidity						116	3.97e+7	0.00–inf	1.00			
Smoker						107	2.35	0.50–11.10	0.28			
Current smoker						17	2.68	0.65–10.70	0.18			
PS1, 2						4	2.40e+6	0.00–inf	1.00			
C–D III												
1-min STST repetition^$^	0.72	0.39–1.00	15	0.97	0.60	144	0.23e–1	1.55e−3–0.17	<0.01	0.35e−1	0.43e−3–0.28	<0.01
Depression of SpO_2_**	0.65	0.284–1.00	4	0.82	0.60	3	1.58	1.12–2.23	<0.01			
%VC	0.73	0.41–0.96	102.00	0.73	0.85	109	0.94e–1	0.01–0.86	0.04	0.15	0.01–1.59	0.1
%FEV1	0.65	0.43–0.86	84.20	0.63	0.80	92	0.15	0.16e−1–1.37	0.10			
Age	0.68	0.59–0.77	74	0.57	1.00	57	7.09	0.77–69.10	0.80			
BMI	0.58	0.26 –0.90										
Comorbidity						116	1.42e+7	0.00–inf	1.00			
Smoker						107	4.19e+7	0.00–inf	1.00			
Current smoker						17	5.78	0.89–37.40	0.06			
PS1, 2						4	6.68e–7	0.00–inf	1.00			

*Number of cases exceeding the threshold or meeting the criteria. **Depression of SpO_2_ was not included as a variable in the multivariate analysis because all repetitions of the 1-MST in the 3 patients were less than or equal to 15. ^$^In the multivariate analysis for C–D II or III complications, 1-min STST repetition was treated as a binary variable (≤20 vs. >20). For C–D III complications, it was treated as a continuous variable, with the odds ratio representing the effect per 1-repetition increase.

AUC: area under the curve; CI: confidential interval; RR: relative risk; C–D: Calvine–Dindo category; SpO2: peripheral capillary oxygen saturation; PS: performance status; 1-min STST: 1 minute sit-to-stand test; VC: vital capacity; FEV: forced expiratory volume; BMI: body mass index

## Discussion

This study suggests that the preoperative 1-min STST has potential as a predictor of postoperative complications in patients undergoing VATS lobectomy. This finding is consistent with a growing body of literature highlighting the utility of simple functional tests in preoperative risk assessment.

Our identified threshold of ≤20 repetitions (AUC 0.70) for C–D grade II or III complications aligns with the <20 repetitions threshold found by Boujibar et al.^8)^ However, their prospective study reported a higher predictive accuracy (AUC 0.85). This difference may be attributable to study design (prospective vs retrospective) and variations in patient populations. Similarly, Quadflieg et al. reported a threshold of ≤22 repetitions (AUC 0.71) in a multicenter study, which further supports a cut-off in the low 20 s for identifying at-risk patients.^1)^

A critical finding in our study is the trade-off between sensitivity and specificity. While the 1-min STST demonstrated high sensitivity (90%–97%), its specificity was low to moderate (46%–60%), implying a high false-positive rate. This combination of high sensitivity and moderate specificity suggests that the 1-min STST is best positioned not as a definitive diagnostic test, but as an effective initial screening or triage instrument. Its primary value lies in identifying a cohort of apparently fit patients who may have underlying functional deficits, thereby warranting a more comprehensive workup, such as a formal cardiopulmonary exercise test (CPET). It can effectively identify a cohort of patients who warrant a more comprehensive functional workup, such as a formal CPET, as suggested by other authors. This approach aligns with clinical pathways where low-tech tests can guide further, more definitive risk assessment. For instance, 1 patient in our initial cohort was excluded from surgery based on a “severely positive” test result (<10 repetitions with significant desaturation), prompting further evaluation that confirmed a very high surgical risk.

Furthermore, the 1-min STST provides complementary information beyond traditional PFTs. As our multivariate analysis showed, a low STST count was an independent predictor of complications, and it was the sole significant predictor for severe (grade III) events. This highlights its unique value. A clinically important scenario is that of a patient with preserved pulmonary function (e.g., normal %FEV1) but poor STST performance. Such a profile suggests that the patient’s risk is not driven by ventilatory limits but by systemic factors such as sarcopenia, frailty, or occult cardiac dysfunction. The 1-min STST, as a measure of overall physiological reserve, is uniquely positioned to uncover this holistic risk profile, which PFTs alone would miss.

### Limitations

Limitations of this study include its retrospective, single-center design and the small number of severe complication events (n = 5), which limits the statistical power of our analysis for this endpoint. This is directly reflected in the very wide 95% CI (0.39–1.00) for the AUC in predicting grade III complications, and thus the findings for severe complications should be considered preliminary and interpreted with caution.

Furthermore, this study did not include preoperative diffusing capacity of the lung for carbon monoxide (DLCO) as a variable. While DLCO is an important predictor of postoperative complications, it was not measured routinely for all patients in our cohort during the study period, and its inclusion in the analysis would have led to a significant loss of data and reduced statistical power.

Additionally, an interesting trend was observed among patients who developed complications, where fewer repetitions appeared to correlate with a larger drop in oxygen saturation (▲SpO_2_). This is physiologically plausible, as patients with lower functional capacity are more likely to have limited cardiopulmonary reserves, leading to more significant exertional desaturation. Although the number of complication events in our study is too small to draw a definitive conclusion, this observation suggests that a composite endpoint combining both repetition count and the degree of desaturation could potentially be an even more powerful predictor of postoperative risk. This warrants investigation in future prospective studies.

Future research should involve larger, multicenter prospective studies to validate these findings and firmly establish the role of the 1-min STST in preoperative clinical pathways.

## Conclusion

The 1-min STST shows potential as a simple, noninvasive screening tool for preoperative risk stratification in patients undergoing VATS lobectomy. However, its clinical utility is tempered by moderate predictive accuracy and low specificity. Before it can be recommended for routine clinical use, its value must be validated in larger, prospective multicenter studies.

## Declarations

### Data availability

The data that support the findings of this study are available from the corresponding author, Dr Noriyoshi Sawabata, upon reasonable request. Due to the nature of this research, which involves sensitive patient information, the data are not publicly available to protect patient privacy and confidentiality, in accordance with the ethical approval granted by the Medical Ethics Committee of Nara Medical University.
